# Clinical research associates experience with missing patient reported outcomes data in cancer randomized controlled trials

**DOI:** 10.1002/cam4.3826

**Published:** 2021-04-09

**Authors:** Michael J. Palmer, Terry Krupa, Harriet Richardson, Michael D. Brundage

**Affiliations:** ^1^ Department of Public Health Sciences Queen's University Kingston ON Canada; ^2^ Cancer Care & Epidemiology Queen's Cancer Research Institute Kingston ON Canada; ^3^ School of Rehabilitation Therapy Queen's University Kingston ON Canada

**Keywords:** data quality, patient reported outcomes, qualitative research, randomized controlled trial

## Abstract

**Background:**

Missing patient reported outcomes data threaten the validity of PRO‐specific findings and conclusions from randomized controlled trials by introducing bias due to data missing not at random. Clinical Research Associates are a largely unexplored source for informing understanding of potential causes of missing PRO data. The purpose of this qualitative research was to describe factors that influence missing PRO data, as revealed through the lived experience of CRAs.

**Methods:**

Maximum variation sampling was used to select CRAs having a range of experiences with missing PRO data from academic or nonacademic centers in different geographic locations of Canada. Semistructured interviews were audio‐recorded, transcribed verbatim, and analyzed according to descriptive phenomenology.

**Results:**

Eleven CRAs were interviewed. Analysis revealed several factors that influence missing PRO data that were organized within themes. PROs for routine clinical care compete with PROs for RCTs. Both the paper and electronic formats have benefits and drawbacks. Missing PRO data are influenced by characteristics of the instruments and of the patients. Assessment of PROs at progression of disease is particularly difficult. Deficiencies in center research infrastructure can contribute. CRAs develop relationships with patients that may help reduce missing PRO data. It is not always possible to provide sufficient time to complete the instrument. There is a need for field guidance and a motivation among CRAs to contribute their knowledge to address issues.

**Conclusion:**

These results enhance understanding of factors influencing missing PRO data and have important implications for designing operational solutions to improve data quality on cancer RCTs.

## Graphical abstract

This qualitative research provides essential inductive evidence for influences of missing PRO data. The results have important implications for designing operational solutions to improve data quality on cancer RCTs.

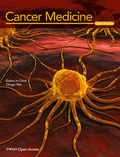

## 1 INTRODUCTION

Missing PRO data threaten the validity of PRO‐specific findings and conclusions from RCTs by introducing bias due to data missing not at random.^1^ Preventing missing PRO data is recommended but may be difficult to achieve without recognizing the influential factors.^2^ A systematic review of the literature identified 46 categories of factors reported to have association with missing PRO data, classified as characteristics of the instrument, patient, center, staff, or study.^3^ Whether each factor has importance is generally unknown, because the strength of evidence supporting each factor was variable.

CRAs are frontline research personnel and a largely unexplored source for informing understanding of potential causes of missing PRO data. CRAs have responsibility for administering instruments to cancer patients on RCTs and processing the data afterward, as within a multicenter cooperative oncology group like the Canadian Cancer Trials Group.^4^ Qualitative research methods can be used to study the experiences of CRAs to gain insight.

The purpose of this qualitative research was to describe factors that influence missing PRO data, as revealed through the lived experience of CRAs. In so doing, we sought to better understand the dynamics underlying missing PRO data and contribute to efforts for improving data quality.

## 2 METHODS

Descriptive phenomenology,^5^, ^6^ as proposed by Caelli, was the methodology chosen. Specifically, the focus was CRAs experience with missing PRO data in context and their perceptions of potential influences leading to missing PRO data.^7^ This practical approach applied to research focused on the reality of CRAs experience as they engaged with the phenomenon through their daily activities.^8^

### 2.1 Data collection

The data collection method was semistructured interviews, conducted in person or by phone.^9^, ^10^ A letter of invitation was sent via e‐mail. Each CRA had opportunity to ask questions before beginning. Participation was voluntary, involving no financial incentives.

The interview guide framed discussion within the CRA's lived experience with missing PRO data, ensuring similarity across interviews. All questions were open‐ended and included prompts to clarify intent and to elicit additional information. The CRA had opportunity to debrief at the end with the audio‐recorder turned off, then provided background and demographic information. Subsequently, the interviewer prepared a field note to capture salient impressions.

### 2.2 Purposeful sampling

Maximum variation sampling^11^ was chosen to select CRAs having a range of experiences with missing PRO data from academic or nonacademic centers in different geographic locations of Canada.

A pool of candidates was created from the CCTG roster, with CRAs having involvement on specific RCTs or membership on disease site committees, supplemented with CRAs having previously participated in our preliminary focus group or by recommendation of a key informant. Candidates were individually invited for interview. Three unsuccessful attempts at contact prompted purposeful sampling of another CRA. Consistent with qualitative research processes, data collection continued until no new themes emerged from interviews suggesting saturation had been reached.^12^, ^13^ Queen's University (HSREB #6015243) provided ethics approval. Written informed consent was obtained from all participants.

### 2.3 Data management and analysis

Each interview was audio‐recorded and transcribed verbatim. The analysis followed Moustakas's modification of the Stevick–Colaizzi–Keen method for descriptive phenomenology.^14^ Bracketing mitigated the investigator's influence.^15^ Recognizing that preconceptions can affect the validity of research using descriptive phenomenology, the investigator implemented a managing strategy to sustain objectivity^16^ by developing a reflexivity statement, reflecting throughout the study, and meeting regularly with a qualitative research expert, thereby ensuring personal assumptions and prior knowledge did not influence the research process. Horizonalization included four steps: identifying relevant statements by considering how each relates to the research question, evaluating the uniqueness of each relevant statement, identifying “invariant meaning units” by removing the redundant statements, then organizing invariant meaning units into clusters and grouping related clusters into themes. Synthesis of findings involved preparing a textual description of CRAs experience with missing PRO data and a structural description of contexts influencing missing PRO data. Verbatim examples were selected to illustrate key information and to summarize the scope of experience.

### 2.4 Rigour and credibility

Sampling ensured that all CRAs had personal experience with missing PRO data on cancer RCTs. Authors performed triangulation^17^ and face‐validity exercises, reaching consensus that each cluster was a factor having relevance to missing PRO data and every theme reflected the associated factors. There was no attempt to discern relative importance.

Refer online for: interview guide, example of field note, summary of the triangulation and face‐validity exercises, a textual description of CRAs experience with missing PRO data, and supplementary tables.

## 3 RESULTS

Interviews, held with 11 CRAs between February 2018 and October 2018 (Table 1), were mostly face‐to‐face and lasted on average 61 min. Two CRAs explicitly declined, because of either another priority or a recent retirement. Analysis revealed several factors that influence missing PRO data that were organized within themes. Table 2 presents the themes and the associated factors. The following sections highlight each theme with examples of factors.

**TABLE 1:** Characteristics of participants.

Geographic location of center	Western provinces of Canada		3
	Ontario		6
	Quebec		1
	Eastern provinces of Canada		1
Type of center	Academic^a^		6
	Nonacademic		5
Worked as a Clinical Research Associate at another centre	Yes		2
	No		9
Worked as a Clinical Research Associate for an organization other than Canadian Cancer Trials Group^e^	Another cooperative group		9
	Industry		10
	Within center		7
	Other		1
Age (years)	<21		1
	21–30		1
	31–40		4
	51–60		5
Education	High school diploma or certificate		1
	Registered apprenticeship or other trades certificate or diploma		1
	College, CEGEP^b^, or other non‐university certificate or diploma		4
	University bachelor's degree		3
	University master's degree		2
Experience with collection of PROs^c^ on RCTs^d^ (years)	1–5		2
	6–10		2
	11–15		4
	16–20		1
	>25		2
Received specific training for collection of PROs^c^ on RCTs^d^	Yes	from a site initiation visit	4
		for an electronic system or device	2
		from a course, seminar or numerous webinars	1
		from case report form guidelines for entering data	1
	No	relied on the protocol with advice from colleagues	2
		relied on self‐education from journals and on‐line literature	1

^a^ Determined by presence of a post‐graduate residency training program in oncology or hematology.

^b^ Collège d'enseignement général et professionnel.

^c^ Patient‐reported outcomes.

^d^ Randomized controlled trials,

^e^ Total exceeds 11 as more than one answer provided.

**TABLE 2:** Themes **(bold)** and the associated factors that influence missing PRO data (**bullets**) identified in analyses of data from Clinical Research Associates (*n* = 11).

**1. PROs for routine clinical care compete with PROs for RCTs.** In many provinces, PROs for clinical care are mandated and used as symptom screening tools. Sometimes patients are not sure why they are asked similar questions in an instrument for an RCT after previously completing an instrument for clinical care. Sometimes patients experience overload and will refuse to complete the instrument for an RCT after previously completing an instrument for clinical care.
**2. Both the paper format and the electronic format have benefits and drawbacks.** Each format has characteristics that can increase or reduce missing PRO data. CRAs have responsibility for entry of data collected by paper and could make a data entry error. The quality and availability of the technology depend on the vendor and may not be apparent when a centre decides to join the RCT. Older patients in particular find technology to be challenging and some will not complete an instrument on a device. The electronic format is not appropriate for some scenarios, such as for follow‐up when the patient stops protocol treatment and is receiving palliative care. The electronic format can evoke an emotional reaction in the patient, is not always the unanimous preference of CRAs, but is thought to be best for future patients. The electronic format may not offer an improvement in compliance over the paper format.
**3. Missing PRO data are influenced by the quantity, repetition, and wording of questions, and by the format of the instrument.** The length of the instrument can lead to missing PRO data. Repetitive questions in an instrument are frustrating for patients. The wording of questions can be confusing or difficult to understand. The content addressed by some questions can be sensitive to some patients. A smaller font size and busy page layout contribute to missing PRO data. Some questions relating to treatment are difficult for the patient to answer at baseline when treatment has not been received.
**4. Missing PRO data are influenced by personal characteristics of the patient, attributes of the cancer, and toxicity from the treatment.** CRAs have different experiences regarding whether age is an influence. Women are typically more likely to skip certain sections, whereas men will often miss a page. Retired people are often more willing to complete an instrument. Some patients have difficulty reading and require assistance to complete the instrument. A caregiver can help, by reading the questions to the patient and recording the answers. Some patients may require use of an interpreter or translator, but if they have to leave, the patients can't complete the instrument. Finding out the assigned treatment was standard of care may make the patient less likely to complete the instrument. Patients with a diagnosis of brain cancer have more difficulty completing the instrument. Patients on adjuvant therapy may be more likely to complete the instrument but miss some of the sections. Some patients are fatigued and don't want to complete an instrument. Some patients develop a mental fog from chemotherapy which can compromise their ability to complete an instrument.
**5. Assessment of PROs at progression of disease is particularly difficult.** The patient's deteriorating health may make them unable to attend the clinic. The CRA may suspect that the patient is progressing but the doctor has sole authority to inform the patient of disease progression. The CRA has responsibility to perform trial requirements and may administer the instrument with bad news pending. Field guidance from the sponsor may encourage the CRA to collect PRO data at progression, but the CRA may not do it (with support of the doctor). The CRA may not be aware the patient has progressed or is not able to administer the instrument before the patient is informed of progression. Being aware of the patient's disease progression may evoke an emotional reaction in the CRA that hinders them from administering the instrument. Being informed of disease progression by the doctor may evoke an emotional reaction in the patient that hinders them from completing the instrument. The patient's personality influences whether the instrument is completed at progression.
**6. Deficiencies in research infrastructure at the center can lead to missing PRO data.** Inadequate space in the clinic to meet with the patient, lack of organization in preparing the chart for the patient's next visit, and priority given to RCTs with higher funding can lead to missing PRO data. Not having sufficient CRAs for back‐up can lead to missing PRO data. Forgetting to administer the instrument to the patient, or not checking whether the patient completed the instrument, can lead to missing PRO data.
**7. CRAs develop relationships with patients that may help reduce missing PRO data.** CRAs develop a rapport with the patients to help them feel comfortable in the clinic. The CRAs are a resource for the patients. The CRAs have empathy for the patients. Frequent visits (such as, during treatment) reinforce familiarity.
**8. It is not always possible to adapt the assessment schedule and to provide sufficient time to complete the instrument.** The amount of time spent with the patient is variable but is less after randomization. The patient has insufficient time to complete the instrument in the clinic because of interruptions. The CRA is busy and is not able to administer the instrument. The necessity for a test or procedure on the day of assessment can make it difficult for the CRA to administer the instrument before the patient is seen by the doctor or receives treatment. The patient has had a test or procedure and, anticipating receipt of bad news, does not want to complete the instrument. Rigid adherence to an assessment schedule can lead to missing PRO data when treatment is deferred but the patient is still expected to come in to clinic to complete the instrument. A delay in treatment, because the patient is not feeling well or blood counts are too low, can necessitate repeating the assessment but the patient may be unwilling to comply. It's time consuming for the patient to complete an instrument and some patients will leave the clinic because they are working or have other appointments. The patient may complete the instrument on a day that was beyond the allowable window for the expected day of assessment.
**9. There is a need for field guidance and a motivation and knowledge base to address issues.** Trials are getting much more complex. It seems like there is more missing PRO data now. CRAs have enthusiasm for cancer research and are concerned about missing PRO data. CRAs wonder about the quality of the PRO data that are collected. The expectation for CRA review of PRO data when collected in a diary may be different from when PROs are collected in an instrument. The contribution of a monitor to reduce missing PRO data at the center is not consistent across RCTs. The CRAs have collective wisdom and can be a resource regarding what can be done to minimize missing PRO data.

We did not find qualitative evidence that sociodemographic characteristics of the CRAs, such as age, gender, or years of experience, to be factors that influence missing PRO data. The CRA's age did not seem to influence administering the instrument at disease progression. However, older CRAs expressed more difficulty with electronic devices and preference for collecting PRO data via a paper format.

CRAs having more than 10 years of experience with collection of PROs on RCTs noted some new staff as being nervous or uncomfortable. However, training made new staff aware of procedures, such as remembering to administer the instrument and check it afterwards for completion. The CRAs learn on the job but this notion of gained wisdom was also described by CRAs with less than 10 years of experience.

### 3.1 PROs for routine clinical care compete with PROs for RCTs

In many provinces, the cancer agency requires all patients to complete an assessment of their symptoms before every appointment with a doctor, but if patients are only attending the clinic to receive treatment, they do not have to complete the assessment.

Sometimes patients are not sure why they are asked similar questions in an instrument for a RCT after previously completing an instrument for clinical care. CRA_G described a patient response “I already answered this question. I already said if that I was tired.”

Patients can experience a sense of being overburdened by what they see as duplication and refuse to complete the instrument for a RCT, as explained by CRA_B: “I frequently hear like ‘arrrgh I already did that questionnaire, I don't want to do that questionnaire.’”.

### 3.2 Paper and electronic formats both have benefits and drawbacks

CRAs identified a trend towards collecting PRO data with electronic devices, while paper is still used for some RCTs. Each format was experienced as having characteristics that can increase or reduce missing PRO data. For example, with paper, there is a risk of a few questions missing, but with electronic devices, always there is a risk of a missing instrument from unreliable technology (CRA_H).

CRAs described their experience collecting PRO data with a tablet, identifying variability in quality of the technology, but also its availability: some RCTs have one device for all patients, or one device for every patient, to use in‐house, while others require patients take the device home and complete assessments for the duration of their life.

CRAs noted older patients in particular as finding technology challenging and experienced some not willing to complete an instrument on a device. There was consensus that using technology for PRO data collection is not appropriate for all patients, but there was no agreement upon the age of patients associated with suitability for technology.

Interestingly, no one described any patient having an emotional reaction to using paper for data collection. In contrast, CRAs described some being frightened by an electronic device and others having enjoyment with new technology. The electronic format was not the unanimous preference of CRAs but was thought best for future data collection, when most patients will likely be comfortable with technology. However, CRA_D expressed concern in the present that these devices cause missing PRO data because patients are not able to use them.

### 3.3 Characteristics of the instrument

CRAs described how the number, repetition, and appearance of questions within the instrument can lead to missing PRO data. In some instruments, the wording of questions can be confusing or difficult to understand. For example, words like “belching” can be unfamiliar to patients (CRA_H). Patients can be sensitive to the content of some questions. For example, those focusing on sexuality were often unanswered.

### 3.4 Characteristics of the patient, cancer, and treatment

CRAs noted personal attributes they experience as influencing missing PRO data including age and gender. With respect to age, CRAs experienced elderly patients with diminished health, such as those with physical limitations or blurred vision from diabetes, as more likely to have missing PRO data. With respect to gender, some CRAs experienced women as more likely to skip questions if they feel uncomfortable with the topic. While men may be less likely to be embarrassed by certain questions, they were experienced by some CRAs as more likely to miss the next page by forgetting to turn the current page after answering the questions.

### 3.5 Assessment of PROs at disease progression

Administering the instrument at the time of disease progression can be challenging for both CRAs and patients. Being aware of the patient's disease progression may evoke sadness in the CRA and feeling like a “jerk” while pursuing something not important to the patient at that time. Similarly, patients when informed of their disease progression can experience feelings that influence their ability to complete instruments. These are emotions CRAs have to negotiate to get the work done.

CRAs noted personality qualities they experience, such as the acceptance of illness, level of commitment, and stress response, can influence whether the patient completes the instrument at progression. For example, CRA_B described two male patients with lung cancer who each received chemotherapy and blinded immunotherapy. At progression, one did not accept his condition and refused to complete the instrument, while the other gladly did the instrument.

### 3.6 Deficiencies in center research infrastructure

CRAs work on many different RCTs. CRA_I described a pharmaceutical company‐sponsored RCT that was more strict requiring high compliance with instrument completion, in comparison to others: “We really have to make sure that the patient fills everything out if that's what they want to do, triple checking and making sure everything's done right.” CRA_I did not elaborate on the funding criteria but conveyed the notion priority given to RCTs with higher funding, can lead to missing PRO data on RCTs with lower funding: “Sometimes it's not possible. We have to trust that [pause] it gets done.”

### 3.7 CRAs develop relationships with patients

CRAs build rapport with their patients so that they feel comfortable doing what is asked of them. CRA_C described doing this for a patient with metastatic pancreatic cancer who attended clinic with her spouse, by bringing items to give them and sitting together, chatting about things like birthdays: “This study, it's not easy, they're in three weeks out of four for chemo. The last time I saw them I said ‘I'll be back next week to see you, see how you're doing.’”.

Being a resource for the patients is an important duty that can involve providing answers to diverse questions, such as resolving scheduling issues, and performing telephone triage for those who are not doing well, such as from immunotherapy (CRA_F).

Clearly, responses suggest empathy for the patients. CRA_D had a patient with bladder cancer experience difficulty completing an instrument and said “You just think oh the poor person.” CRA_I described how treating a single metastasis with radiation can often delay progression, but for one patient “it came back [pause] uhm much earlier than expected. It was kind of sad.” This empathy highlights sensitivity of CRAs when collecting PRO data.

CRAs strengthen their relationships while administering instruments, such as during treatment, getting attached to the patients, and sometimes, observing their passing. CRA_E vividly recalled one patient who, after reacting to chemotherapy and having no family, requested company while dying.

### 3.8 Time available for assessment

CRAs described many challenges with administering the instrument. Among these challenges is providing sufficient time for the patient to complete the instrument, which may be difficult because of interruptions. This seemed to be more frequent in academic centers, where many people were involved in the circle of care. First, the patient is put in a room. Next, a nurse enters and does a clinical assessment of the patient. Often, medical students or a resident enter and perform their assessment of the patient. Finally, the doctor enters to meet with the patient. Accordingly, the CRA has very little time to get the patient to complete the instrument because of frequent interruptions.

### 3.9 Need for field guidance

CRAs offered suggestions regarding what can be done to minimize missing PRO data. Several CRAs recommended improvements to the instrument. Including the time frame such as “in the past seven days” in each question rather than providing it at the beginning of a group of questions would remind patients to answer each question. Asking only essential questions, rather than asking several questions about the same thing in different ways, would help minimize stress on patients. Creating a cohesive instrument with everything well‐spaced would reduce missing PRO data. Many suggestions were made related to how structures and processes could be put in place. These include providing training, having time and space to complete the instrument, and allowing flexibility with the schedule of assessment so that CRAs have more opportunity to collect PRO data from the patients. Organizing the documents in a plastic sleeve at the front of the chart was noted as a practical way to keep the documents together so that they are not missed.

## 4 DISCUSSION

Missing PRO data are a serious problem that limits achieving high‐quality PRO data on cancer RCTs. To better understand what may cause its occurrence, we interviewed 11 CRAs, obtained descriptions for factors that influence missing PRO data and organized these factors within themes. Their lived experiences enhance our understanding of missing PRO data as a multidimensional and complex phenomena.

We are confident that each factor had relevance to missing PRO data and every theme reflected the factors aligned with that theme. Of note, we did not attempt to discern relative importance of these factors. Any change involving a factor should lead to better data from PROs, but the extent of impact is not known.

Our study was conducted before the COVID‐19 pandemic which prompted changes in practice, and as additional causes of missing PRO data may now exist, highlights the need to better understand the underlying dynamics so that the system can adapt to new stressors without increasing risk of missing PRO data.

Typically, collecting PRO data on a RCT involves the patient being given an instrument before randomization, indicating their answers to the questions, and returning it, repeating this action at subsequent time points during treatment and follow‐up. While the process appears straightforward, there are inherent complexities. It is apparent that differences—both within and across the instruments, patients, centers, staff, and RCTs—contribute to making the collection of PRO data challenging.

PROs for routine clinical care, such as ESAS^18^ and EPIC‐CP,^19^ can help health care practitioners improve the quality of clinical care for cancer patients by screening symptoms, routinely expected before an assessment for an RCT. This finding raises awareness that, in many provinces of Canada, there is an expectation of patients—external to and independent of their participation in a given RCT—which may lead to missing PRO data on the RCT through patient uncertainty or overload.

Surprisingly, qualitative inquiry of research staff has not commonly been used to identify influences of missing PRO data. In the systematic review of the literature,^3^ one study from the United States^20^ used a focus group to identify issues with retention. Subsequently, investigators from England^21^ and Australia^22^ used interviews to obtain current practices for identifying missing PRO data and procedures for following up. Our interviews with CRAs in Canada demonstrate CRAs have insight for influences of missing PRO data. For example, direct evidence from CRAs that PROs for routine clinical care compete with PROs for RCTs has not been previously reported to our knowledge. Also, our results contribute additional evidence to the existing literature for several factors involving characteristics of instruments and patients, research infrastructure, and data capture format.

Published guidance exists for improving the quality of PRO data. The documents do not provide operational guidance for CRAs in dealing with missing PRO data but instead guide researchers with writing the protocol,^23^ analyzing the data,^24^ reporting the results,^25^ and graphically displaying the findings in clinical practice.^26^ Collectively, these are useful to maximize PRO results on RCTs,^27^ but despite careful planning, missing PRO data will occur. Minimizing missing PRO data is possible, by first understanding potential influences and then applying complementary strategies to reduce negative consequences.^28^ Guidance on how to do this in the field would help CRAs, who feel accountable for data quality, achieve high‐quality PRO data on cancer RCTs.

A main strength of the study is the inclusion of several measures^29^, ^30^ to establish rigor and credibility. Saturation, the extent data are comprehensive and provide a range of perspectives, was achieved when no new themes emerged after 11 interviews.^31^ A main limitation is CRAs may have withheld information. One CRA said after the interview that they would not admit making an error. The effect of the audio‐recorder on content is unknown.

This qualitative research provides essential inductive evidence for influences of missing PRO data. Given these results, a pertinent question is: How best to move forward to affect change? Our findings suggest focus should be on developing field guidance for CRAs, which is a practical strategy towards prevention of missing PRO data. CRAs have motivation to contribute their knowledge and should be involved. Unlike the current set of documents, some of the guidance content may be specific to cooperative oncology groups, or academic versus pharma RCTs but needs exploration. As well, it may be feasible, in the future, for CRAs to directly extract data from PROs completed for routine clinical care and meet with the patient to collect any missing information or additional PROs for the RCT.

In conclusion, these results enhance understanding of factors influencing missing PRO data and have important implications for designing operational solutions to improve data quality on cancer RCTs.

## CONFLICT OF INTEREST

The authors declare that they have no conflict of interest.

## Supporting information

Supplementary Material

## Data Availability

The data underlying this article contributed to a dissertation which is subject to an embargo through Queen's University. Once the embargo expires, the data may be available upon reasonable request from the first author.
