# Supplementary material for: Clinical research associates experience with missing patient reported outcomes data in cancer randomized controlled trials
Source: Cancer Med. 2021 Apr 9;10(9):3026–34. doi: 10.1002/cam4.3826 (PMC8085912; doi:10.1002/cam4.3826)
Supplement: Supplementary file 1 — Supplementary Material [file CAM4-10-3026-s001.docx]

Supporting Information

**Clinical research associates experience with**

**missing patient reported outcomes data in cancer randomized controlled trials**

***Cancer Medicine***

**Michael J. Palmer PhD**, **Terry Krupa PhD**, **Harriet Richardson PhD**,

**Michael D. Brundage MD MSc**

**Corresponding author**

Michael J. Palmer PhD, Cancer Care & Epidemiology, Queen’s Cancer Research Institute, Kingston, ON, Canada K7L 3N6

Email: [81mjp6@queensu.ca](mailto:81mjp6@queensu.ca)

The Supporting Information provides: Interview Guide for Clinical Research Associates, Example of Field Note, Summary of the Triangulation and Face-validity Exercises, A textual description of CRAs experience with missing PRO data, and nine supplementary Tables.

Interview Guide for Clinical Research Associates

| Heading | Content | Rationale |
| --- | --- | --- |
| Preamble  Start | I would like to ask you some questions. I will listen to what you say. I will make a few notes but please continue to talk.  Are you ready to begin? Okay, I will turn on the audio-recorder. | To focus attention |
|  |  |  |
| Behaviour | 1. I’m interested in your experience with asking a cancer patient to complete a PRO instrument on a RCT. What is a typical encounter like? Take me through the steps.  Prompt:  Describe the usual process. | To initiate CRA thinking about the process and to obtain information on how it works. |
| Experience | 2. Tell me about some of your memorable experiences with missing PRO data.  Prompt:  Take your time. Reflect on your experiences. | To capture what the CRA experienced with missing PRO data, for the textual description, and to capture the contexts in which the CRA experienced the missing PRO data, for the structural description. |
|  | 3. Can you share one or two stories about missing PRO data?  Prompt:  Think of your own practice and describe an experience with missing PRO data that was meaningful to you. |  |
|  | 4. In your experience what are the important influences leading to missing PRO data?  Prompt:  What are the things that most influence missing PRO data?  What situations especially influence missing PRO data?  What influences do you feel are the most important? |  |
|  | 5. It has been said that the important influences leading to missing PRO data may depend on whether it is before or after randomization. I am interested in your experience. Can you give me one or two examples?  Prompt:  Think back to when an assessment was expected but the patient did not complete all or part of the instrument. It may be at baseline, or it may be during treatment or follow-up. Describe your experience with the missing PRO data.  Have you had experience with each of these? |  |
|  | This next section is about a number of things. I will ask you about each of them, one at a time.  6. Tell me what your experiences are as to whether or not instruments influence missing PRO data.  Prompt:  Quality of life questionnaires are examples of instruments. Think about your experience with the instruments. Was there anything about the instruments that contributed to missing PRO data? Tell me about it.  7. Tell me what your experiences are as to whether or not cancer patients influence missing PRO data.  Prompt:  Think about your experience with the cancer patients. Was there anything about the cancer patients that contributed to missing PRO data? Tell me about it.  8. Tell me what your experiences are as to whether or not the centre influences missing PRO data.  Prompt:  The centre is the facility where you work as a CRA. Think about your experience with the centre. Was there anything about the centre that contributed to missing PRO data? Tell me about it.  9. Tell me what your experiences are as to whether or not the staff influences missing PRO data.  Prompt:  The staff is research personnel working at the centre. Think about your experience with the staff. Was there anything about the staff that contributed to missing PRO data? Tell me about it.  10. Tell me what your experiences are as to whether or not the RCTs influence missing PRO data.  Prompt:  The RCTs are phase III studies. Think about your experience with the RCTs. Was there anything about the RCTs that contributed to missing PRO data? Tell me about it. |  |
| Final question | 11. Is there anything else you would like to tell me about your experience with missing PRO data?  Prompt:  I have no more questions, but I’d like to give you opportunity to share anything else. | To provide opportunity for the CRA to contribute more information |

Example of Field Note

Today, at 2:00 pm, is my second interview. I have never met B, and look forward to interviewing her.

In preparation, I reviewed the interview guide, survey of background and demographic information, and consent form. The audio-recorder is functional and has a fresh battery. The small meeting room is booked and I will post a sign (Interview in Progress) on the door.

I had the following e-mail exchange with B, when scheduling a convenient time and date for the interview:

B: I am sorry I don’t really understand what you are looking for, is it from my perspective, personal experience?

P: I am interested in your own experience. I will ask you to describe your experience with cancer patients. I will be interested in learning about your experience with a few patients who participated on a randomized clinical trial but didn't answer all of the questions in a questionnaire, or didn't complete the questionnaire at all.

B: Thanks for the info, that gives me more of idea of what you are looking for and I can think of patients in these 2 situations prior to our interview. I do not have a lot experience with the paper PRO CCTG studies but I do with ePRO data using a tablet for various pharmaceutical studies I work on. So they can’t miss questions on the ePRO since it won’t let them go to the next page. I have had patients decline during the study for a few reasons or have me work on them with them since they aren’t great at reading or working with electronic device.

My focus is on B and her lived experience with missing PRO data. Given the above information, it will involve EDC. In the previous interview, A described her experience with EDC so it will be interesting to hear the experience of B. Clearly, there is opportunity with B to illicit depth in responses about missing instruments with EDC and, as per RHBS-932 note of 2015FEB19, I will keep a Devil’s advocate question in my back pocket:

‘In the experience of some people, modern technology is so good that with EDC, there are no missing questions because the patient has to answer everything on the screen. However, the experience of other people is that the technology is so bad that with EDC, there are lots of missing instruments. What is your experience?’

I acknowledge my past work within CCTG and at McGill University, and current research observing a focus group of CRAs, conducting a systematic review of the literature and creating a classification framework, and analyzing data from a few RCTs. However, I will not think about what I may know regarding the association of factors with missing PRO data, nor will I think about responses provided by A. Rather, I will actively listen to what B says during the interview. B has expertise. I don’t, and can learn from B’s description of meaningful events.

I met B in lobby and walked to Level 2. B asked if I had previously worked at CCTG, and if one of my RCTs was MA17. Yes. B previously worked in KGH, screening patients with asthma to see who was eligible for a new treatment (no involvement with PRO on RCTs), then transitioned to cancer (experience with PRO on RCTs). B thought I may be ‘Ian’ at CCTG and wondered if purpose was to find out why PROs aren’t administered/completed. No – I have no affiliation with CCTG and purpose of my research is to understand why PROs are sometimes missing on RCTs.

Provided overview, obtained informed consent, asked if B had any questions (no), turned on audiotape and began interview at 2:10.

1. Good overview of ePRO with tablet, visits and baseline and after randomization. Sometimes the tablet contains 3-4 questionnaires.
2. Lung cancer. One patient: elderly, old school, 70 years. Another patient: different trial, B reads questions and records his answers, 60 years. Compared with paper, with tablet, answer all questions.
3. ESAS – completion mandated for all patients, done electronically in clinic before ePRO on tablet or cell phone, but if ESAS not done, paper copy provided in chart outside room. PRO-alert described. Patient burden: same questions asked.
4. Lung cancer: PRO after progression. MERCK blinded study: stop at safety visit. Assessment at discontinuation. Two males had progressed, were unblinded, had both received treatment not placebo, so study treatment was not an option. One man: angry, upset, would not do PRO. Other man: accepting, would do PRO. Only difference was pathology (squamous versus non-squamous): not reason for not doing PRO.

Lung cancer patients are accepting of their diagnosis (smoking). Breast cancer patients are harder on B personally as asked different PRO questions: body image, attractiveness, sex life, “those questions”, “what kind of pervert would ask those questions?”. One woman with breast cancer would answer paper questionnaire but would refuse to answer group of questions starting Q.41. Missing questions with paper but not tablet. I played devil’s advocate. In her experience with tablet, no missing questions because tablet works. Depends on vendor.

1. Not asked.
2. Length. MA32 – metformin study in breast cancer. Really long questionnaire and dietary questionnaire. Too much. 20 pages.
3. Stage. Adjuvant – highly motivated, more likely to complete PRO. Metastatic – more of a problem completing.
4. No.
5. Sometimes forget to administer questionnaire or forget to provide access to tablet for back-up CRA. Personally embarrassing.
6. Protocol. Discontinuation.
7. Patients get asked so many questions.

Turned off audiotape at 3:30 pm.

B said that stress was not experienced, and no question was inappropriate. However, she was surprised the interview was audiotaped. She did volunteer, with audiotape turned off, that her job involves front-line work with real people who may be dying of cancer. Her boss had an in-house session reminding everyone of importance of collecting urine sample, PRO and other information from all patients even when palliative. B asked how they could do this and said, in some situations and with some patients, she will not do it. She has been in room, crying with a patient who was coping with bad news, and takes this home with her. Lung cancer patients often deteriorate quickly and B is aware of how each patient is doing. Sometimes she can’t ask a patient to complete a PRO. The role of a CRA involves humanity and an emotional burden. I commented I have no clinical experience, but wonder about the dual responsibility to care for a person and to collect data.

B completed survey on background and demographic information. She pointed out her accreditation is ACRP,CCRC which is competitor of CCRP. I said I would revise Q.5 to include this as option.

End 3:40 pm

General impressions:

A very good interview. Articulate, excellent examples, informative. Good description of EDC. B likes tablet and no body language with arms crossed. B laughed and smiled on several occasions. I recall one occasion where she changed from description of patient who had been informed of progression, to a similar patient who completed questionnaire. I was better at following the discussion, but could improve my formulation of questions. I especially fumbled trying to articulate Q.6.

After about one hour, the first page of the interview guide had been covered. B asked, if I was going to interview more CRAs, she could connect me with some of her colleagues. I explained I was going to interview 20 CRAs in total, all experienced with cancer patients and PROs on RCTs, from different centres across Canada. I asked B if she was comfortable with a few more questions – yes, so I turned the page and asked Q.6 – Q.10. Moving forward with interviews, I should consider not asking these questions if the influence of (instruments/patients/centre/staff/RCTs) have been described in depth previously.

Additional information:

B sent me an e-mail (entitled ‘Interview’) after the interview with the following text and an enclosed figure.

B: It was nice meeting with you last week. As discussed I am sending you a copy of the ESAS questions that patients answer/rate when they come into the cancer center. Below is the electronic print out they receive from the machine after they complete the questions at the kiosk. It shows how they previously have answered at visits. If they miss this, the nurse asks them to complete a paper copy and someone inputs that information so it appears on the print out at their next visit so each visit can be compared. If a patient rates high the nurse is to document her conversation about that particular symptom as to why it is high so help can be offered. Let me know if you have follow-up questions, I would happy to answer.

P: I appreciate receiving a copy of the ESAS questions and additional information about it - this is very helpful!

  I will contact you if I have additional questions.

It was a pleasure to meet you and I learned a lot from the interview!!

Best regards and many thanks,

Summary of the Triangulation and Face-validity Exercises

P and K performed an investigator triangulation exercise by independently identifying relevant statements in the first five interviews then meeting to talk about it, including thoughts on organizing initial clusters in a meaningful way. P brought the next level of analysis to co-authors with the supporting data and discussed emerging themes. R and D expressed confidence in the ability of P to identify clusters from the data.

K, R and D performed a face-validity exercise. For each theme, participants were asked: Is the theme an accurate reflection of the clusters of meaning statements? Is the wording of the theme clear? For each cluster, participants were asked: Is the cluster relevant to missing PROs? Is the wording of the cluster clear?

Participants independently reviewed every theme and the associated clusters of meaning statements, indicated their choices, and provided insightful comments. P collated the responses. A ‘No’ response and/or inquisitive comment prompted P to flag nine themes and 26 clusters for review. P carefully considered the provided information, checked the supporting data as required, and made revisions. One theme was revised to more accurately reflect the clusters of meaning statements and to improve clarity, eight themes were revised to improve clarity, and one theme was kept as is. Twenty-six of the 64 clusters were flagged for review. Two clusters were deleted (because of redundancy and data do not support a link to missing PRO data), two clusters were moved to another theme, and 20 clusters were revised (to improve relevance to the theme, relevance to missing PRO data, and clarity). Two clusters were not changed because the statements accurately reflect the data. Minor suggestions to improve wording were accepted for 13 clusters, and 24 clusters were kept as is.

Participants were provided with a detailed summary of the previous round, and the revised wording in track changes for every theme and associated clusters, clean wording, and a few notes clarifying some of the changes. Participants were asked to review the revised wording and to provide comments if further revision was required.

Participants independently reviewed the content and provided insightful comments. P collated the responses, carefully considered the provided information, checked the supporting data as required, and made revisions. One theme and eight clusters were flagged for review. In the ‘Resource’ theme, two words were deleted because of redundancy. In the ‘Manifested’ theme, four clusters were collapsed into one cluster to simplify wording, and another cluster regarding emotional reaction was kept as is because the supporting data were insufficiently rich to justify creation of a new theme. In the ‘Format’ theme, one cluster was revised to improve clarity. In the ‘Instrument’ theme, one cluster was revised to improve clarity, and a new cluster was added.

Consensus was reached on the final themes and on the final clusters of meaning statements.

A textual description of CRAs experience with missing PRO data

Missing PRO data were an infrequent experience. CRAs explained having very good success collecting PRO data, but having missing PRO data was unusual.

CRAs described different types of missing PRO data. CRA_A described how the entire instrument was missing when a patient refused to complete them during treatment. CRA_G explained the instrument can include several questionnaires with each containing a separate face sheet that the CRA completes and sometimes, the presentation format may prompt the patient to think they have completed the entire assessment and miss some questionnaires. Several CRAs experienced a missing page because the patient flipped pages improperly while going through the instrument. All experienced missing responses to specific questions, recalling patients reading quickly or forgetting to answer.

The missing PRO data can precipitate negative feelings in CRAs who take this responsibility seriously. CRA_E described coordinating a scheduled assessment: ‘it’s very frustrating – CRAs get them back and they’re not completed’. CRA_F said ‘It really feels terrible to forget one of those questionnaires. I’ve really missed sometimes’. CRA_G replied ‘We always feel bad when it happens (laughs). It is never intentional’.

Collectively, the missing PRO data can occur in different patterns and may evoke an emotional reaction in the CRA. These reflect the competence and dedication of CRAs who described their lived experience with missing PRO data from cancer patients on RCTs. CRAs care about data quality and feel accountable for its completeness.

**Table_1.** Each format has characteristics that can increase or reduce missing PRO data.

| Characteristics that can increase missing PRO data | |
| --- | --- |
| Paper | Electronic |
| Sometimes patients don’t complete all the questions | Need to charge |
| Can provide two answers for the same question | Have to load them |
| Not able to provide comments to sponsor | Had problems downloading |
| Having to spend time photocopying | Are not reliable |
| Photocopying error | Don’t have a backup |
| Preserving anonymity | Need wireless connection |
| Sending it off to the data people | Assessment takes more time |
| Can go missing | Finicky to touch |
| Having to keep a copy | Having to keep a copy |
|  | Hard for patients |
|  | There are different procedures for Canadian patients |
|  | It is possible for a patient to provide data for one of multiple questionnaires on the tablet |
|  | Have to use disinfectant |
|  | Need to have a code |
|  | Missing can affect the whole study |
|  | Flow of administration is different and more cumbersome |
| Characteristics that can reduce missing PRO data | |
| Paper | Electronic |
| Availability | Can’t miss any questions |
| Don’t need to charge | Cannot provide two answers for the same question |
| Can move information on the cover sheet | May prompt assessment |
| Can record comments | Don’t have to print documents |
| Can put several on patient’s chart | Less paper in charts |
|  | Don’t have to enter data |
|  | Data automatically go to the sponsor |
|  | Getting e-mails from the sponsor |
|  | The system can flag when health utilities has been missed |
|  | Keeping track of patients in a web-based system |
|  | Have to give it to the patient physically |
|  | CRA is not supposed to see the answers |

**Table_2.** CRAs describe their experience collecting PRO data with an electronic tablet.

| CRA | Quote |
| --- | --- |
| A | The bane of our existence right now are these devices. I find that a big hindrance right now is these electronic version of that. That’s a big mistake, common mistake I would say now. They’re nothing but headaches the electronic devices I find. They’re frustrating. |
| B | The electronic, prior to going onto the trial, I have to register myself an account, whoever else is my backup for the trial, generate accounts. I log in with my account. We screen a patient. I add that patient to the e-PRO, randomized onto the trial. Prior to every visit I log in, activate that visit for the patient, I turn it over to them. |
| D | We’ve had some studies using electronic tablets or handhold devices. They’ve pretty much been disasters (laughs). |
| E | On one study that we do, that’s advanced breast, that’s a drug company one . . . it’s on a laptop. |
| F | The research part of it the quality of life to administer either on a PRO tablet on an e-PRO tablet, six or seven studies. We’ve had them for five six years different studies with uhm tablets. It seems uhm [pause] less personal. The electronic device is more the problem. |
| G | umhum! yup! yup  [Response to And were all the patients on that study expected to complete an instrument with a tablet?] |
| H | We have a tablet in the hospital for some studies. It’s basically like a paper questionnaire but on a tablet. It’s fairly easy to complete. |
| I | We can use the iPad here in [name of city deleted]. We do have a system where certain people get are given uhm an iPad to report . . . on the radiation side . . . a pilot study, several main questionnaires . . . for the GU patients, some head-and-neck questionnaires. It looks like a nice way to uhm collect that right now. |
| J | Newer studies are, yes. We have two of them. Breast study, an oral uhm drug, do uhm the quality of life questionnaire on a **tablet**. They do it uhm [pause] not at every [pause] visit but at every third visit [pause] uhm for the breast study. Prostate study, they complete it using the tablet, a little bit easier. It’s depending on the type of tablet used. It’s also uhm important to note that there are [pause] I think better ones (laughs). |

**Table_3.** Older patients in particular find technology to be challenging and some will not complete an instrument on a device.

| CRA | Quote |
| --- | --- |
| A | We have prostate patients that come in that are in their eighties that are trying to figure out these tablets. Some of them will navigate it but they’ve never dealt with the piece full . . . We’re asking them to take these tablets home and do these diaries and these PROs on these tablets. People that have never been exposed to an iPad or a tablet or those things that it really is a foreign type thing to have to use a device like that . . . You’ve got to touch, give an extra press [sound of finger hitting table several times] They jam at it or their fingers seem to be too big for the little button. I don’t like it. I saw them. I don’t even know how they would do the little tiny phone cell phone like pad, get frustrated with the touch screen inside of it, as do I. |
| B | He’s elderly, he wouldn’t have a cell phone, he’s just old school. He has to tap the button. For whatever reason, I don’t know if he’s using his fingernail, a lot of them use their fingernail [sound of finger hitting table several times] it’s like “no no no you’ve got to use your finger”. It’s just not very sensitive. He gets kind of frustrated because it’s not registering his answers . . . It’s just like a tablet, but they give you a stylus pen, that works okay. I tried giving him one of this stylus pen, that didn’t seem to really help him any, he just kind of struggles through it . . . Some of the older, it’s a little bit overwhelming for them. |
| D | Older patients can’t grasp it. You’ve got to look at your demographics. It’s not so good for older ovarian cancer ladies, prostate men. |
| F | Some of them have never used an iPad because they’re in the older age. 60 and lower take the tablet, they can handle that, they’ve used iPads and iPhones and stuff. The younger ones are more comfortable. The tablets are very simple. There’s that nice big blue arrow they know they have to touch it. They touch every little tab. They understand. The older ones are not as comfortable. |
| G | An older lady, in her late seventies, a hem(atology) patient, CLL <Chronic Lymphocytic Leukemia> trial. She just hated that (laughs). She didn’t want to do it. She just didn’t like it. She sometimes either flat out refused or would skip through. It was technology that she wasn’t comfortable with. |
| I | I’ve found a difference when uhm when I was doing the SPROUT sys(tem) the SPROUT system for SC24. There was an option to go online and even a scan option. The older patients didn’t really follow that, they did not like that, it’s too advanced for them. They didn’t even know what the the scan bar code what that means. If we do convert to a system that [pause] allows [pause] patients to either go online and login and type in an an address and type in PINs and things like that they won’t [pause] there may be a [pause] uhm [pause] you know a difference in age. I feel the younger generation are more comfortable doing. The older generation would probably prefer the paper version. That had the most issues with [pause] the computer system was I think fifty-five and above. |
| J | Our elderly patients that are seventy eighty years old are not used to the electronic [pause] devices. They may you know “give me a newspaper and a pencil” (laughs). There might be some intimidation with how to complete the quality of life electronically. They do complete it. There’s just a lot of anxiety there for the elderly when it comes to electronic devices. It is quite a uhm concern for the [pause] the patients . . .The concern is that it’s some cancer patients and the age. They are more prone to the paper and less to any electronic devices. |

**Table_4.** The electronic format was not the unanimous preference of CRAs.

| CRA | Quote |
| --- | --- |
| B | I don’t have any problems with [the electronic format] myself. I find it very helpful. I do! I like it. It is good! That’s my preference anyway. |
| D | I’m not a fan of the electronic PROs right now. I like paper. It works. Unless you don’t have a pen (laughs). |
| H | There’s still paper. There’s this company actually who brought back to papers. Some sponsor are going back to paper. I think for now I prefer paper because with a device we often have issue, especially when we try to connect. The best would be to have a uhm something that we can connect in the computer [pause] to update the device so it’s ready for the visit. Right now we always have to rely on the Wi-Fi or GSM <Global System for Mobile Communications> to connect [pause] and right now I think this is the weakest. |
| I | The reason why I like the iPad is because I have to program it ahead of time, and then I give it to the patient physically, and then they fill it out, and then I can check afterwards that it’s been sent off, and then I’m done. |
| J | I enjoy the paper. |

**Table_5**. The wording of questions can be confusing or difficult to understand.

| CRA | Quote |
| --- | --- |
| A | They’re wanting to answer them but they just, they get frustrated with the types of questions that are being asked. I think it’s the way they’re formed . . . “What is your best [pause] health today?” throws people when they’ve been told they’ve got an incurable cancer. I think that almost is like a slap in the face to them, **whereas** other people would read that question and have you know record their (answer). Some people read that sentence or will read a sentence and get hung up on the way it’s posed. That’s what makes them not answer it, or not want to answer it, or have a hard time, or ask me for how they should answer it. |
| B | I always hate this one too. It’s like a scale “How would you rate your health today?” 100% is best imaginable and zero is worse than imaginable. People say “Well how do I know what worse than imaginable is? I guess I’d be dead!” (laughs) |
| E | Because they’ll go “how am I supposed to answer that like today I feel”, no no it’s not today it’s in the last seven days or two weeks. |
| H | Language can be a barrier even if the patient always been speaking English or French. Sometimes the language used is not what they currently use at home. They’re not sure what it means or how to answer to it . . . That could be an issue. Once in a while we have patients who really don’t understand some of the words, “belching”, that was one of the words. There was another one in French but now it doesn’t come to my mind (laughs). The patient was speaking French but then read the sentence. She couldn’t remember what was the uhm the meaning of that word. |
| I | There’s a section that talks about pain. One of my patients always fills out “I have pain”. Because it’s such a general question about pain he answers to what he believes is you know general pain. He was saying “well it’s not related to this study and I’ve always had this pain”. He always writes us a note on the side “it’s from an ankle injury years ago”. This happens every time . . . He’s wondering “am I supposed to relate this pain specifically to the area of treatment?” or “am I supposed to be just answering as in general which is the ankle pain I’ve had since uhm since uhm you know for twenty years because I had a I was playing some sort of sport and we were injured?” |
| J | Something to be said for the type of quality of life questionnaires. Have to be the simple ones [pause] uhm nothing too elaborate or or difficult for them to understand. |

**Table_6**. The content addressed by some questions can be sensitive to some patients.

| CRA | Quote |
| --- | --- |
| A | There’s also certain parts of the questionnaire that are commonly left out, specifically breast cancer uhm patients whether, about their sexuality. We have some patients who just will skip that section altogether. They’ll have done that for the entire time they were on a trial. They just will have no interest in sharing that information with us. |
| B | You have mastectomy or lumpectomy, it changes your appearance, your body image, how you look at yourself. A lot of those questions are tapping into that, asking you “do you feel as feminine as?”, “how do you feel attractive?”, asking them about their sex life. I just think those questions are just terrible. A lot of patients that I would administer them they just hated doing them for that reason. One lady she’s like “what kind of pervert wants to know this information?” (laughs) It’s very intrusive . . . I had a patient who, she absolutely refused to answer any of those questions. She’d answer all the other ones . . . it was just this one section when it got into body appearance, how many times you’re having intercourse, with or without a partner . . . I wouldn’t feel comfortable answering those questions either. That would be a reason why you’d be missing that particular data was patients just don’t feel comfortable. |
| C | I suppose an example would be males, when it comes to impotence. Some of the questionnaires are asking those questions, their sexual behaviours. |
| D | Some questions can be sensitive and personal, some people aren’t comfortable divulging that, especially prostrate patients, their sexual function, sometimes they’re embarrassed by it, they don’t want anybody to know that they’re struggling. One lady, an ovarian study, always refused to answer one question. She’d say to me “I don’t know how to answer this. I don’t know what they’re asking”. The question was “does your treatment make you feel less of a woman?” |
| E | There’s no particular questions they miss unless it’s a question that sexuality don’t like to answer that. Don’t need to, can leave that blank if they don’t want to answer it . . . Men don’t seem to care at all about those questions. I find men more open about that than women when they ask the certain questions about their sex life or whatever. |
| F | They will think twice if they want to talk about it if it’s cancer of the prostrate, “do you feel fulfilled sexually” . . . He was married. He says “so uhm if I don’t have to answer that question I won’t”. He didn’t answer. |
| H | . . . if they’re satisfied sexually and things like that. Some patients do not want to answer these questions. |
| I | There’s some questions that of course they don’t like answering . . . |

**Table_7**. CRAs have different experiences regarding whether age is an influence.

| CRA | Quote |
| --- | --- |
| C | We find that younger patients, if they’re on an oral medication, are a little less compliant than older people (laughs). |
| D | A young cervix cancer patient . . . she was okay answering the very personal questions . . . whether that has to do with the age difference. Prostate patients are generally older. A young woman, I don’t know if that mattered, it could. |
| E | I didn’t find a trend, why people miss questions or miss signing, unless they’re really elderly . . . now I would say probably 80 and over. |
| F | My patients are over 60 65. A lot of them are diabetic. They have that blurred vision. There’s a lot of different factors. |
| H | The seventies are new fifty because [pause] the population [pause] is a bit uhm is better fit. Even at seventy years old we have patient you would never think are seventy [pause], they look very fit, they look in shape. It really depends on the condition of the person. Uhm, right now, no – compare my older and younger patients [pause] it’s about the same. |
| I | When it comes to the paper version that we’ve been doing it doesn’t really matter. |
| J | Population is elderly patients. The average is approximately like seventy years old. Anything younger than that is actually a young patient for us. (laughs) Working with many eighty year old patients, sometimes is difficult to complete the questionnaire for them, struggling to just [pause] not necessarily understand the question but physically just doing the questionnaire. |

**Table_8.** Being aware of the patient’s disease progression may evoke an emotional reaction in the CRA that hinders them from administering the instrument.

| CRA | Quote |
| --- | --- |
| A | I sometimes will even gauge going in there. If I went in and she was weepy I wouldn’t even bring my tablet . . . |
| B | The physician goes in and says “unfortunately your cancer is progressed and we’re going to take you off of this treatment”, talks about other treatment options, or there is no treatment options. Obviously, it’s very emotional. I’m to come in and ask them to do a discontinuation e-PRO at that point. Sometimes they handle that information very well. I’ve had patients too upset. I feel like a bit of a jerk, too. “Hey I’m really sorry yeah and you’re off the study and da da duh but the study would really like me to have you do this questionnaire how do you feel about that?” They’re like “no”. |
| C | . . . they progressed, they’re not feeling well, they may not want to complete the QofLs. If you look at a lot of the studies, once they do progress, QofLs aren’t needed any more. Perhaps that’s why, because would I want to chase a patient for that? That would be the last thing I would probably want to chase them for. At that point that’s not important to them. It is to the study, but not to the individual. |
| D | . . . umhum [spoken very quietly] yeah I’m sure I have [long pause of three seconds] it is difficult. “I I understand this is a a hard time, you’re able to refuse any study procedures you’re not comfortable with, but would you complete this today or” |
| E | Oh, I get very emotional with them, too. It bothers me when they’re upset. |
| F | It doesn’t. [Response to How does that affect you?] I mustn’t felt very good because I asked him if it’s okay [pause] if we could do it together. I must not have been satisfied that he didn’t do it at all because I wanted really to know how he felt. |
| G | We don’t even [pause] uhm [pause] typically we wouldn’t bother them with that [spoken very slowly] [spoken very quietly] at that point yeah [spoken very quietly]. |
| I | It’s very [pause] very sad. It’s [pause] not a great [pause] great position to be in. It makes me s(ad) yeah it makes me kind of [pause] you know sad as well. I approached him with it. I tell them like “I really understand if you don’t want to do this” so [pause] “I can I can always [pause] see you the next time” or something like that [spoken very quietly] I think they did end up doing the next one. |

**Table_9.** The patient’s personality influences whether the instrument is completed at progression**.**

| CRA | Quote |
| --- | --- |
| B | I think it’s just personality. I just feel like some people have a different handle on their cancer, their diagnosis, how they accept it. It’s a difference in personality I think . . . I recently had another patient, exact same design as that trial but a different type of lung cancer . . . progression . . . found to be on the immunotherapy, on the clinical trial drug . . . He handled it very very well . . . He was obviously upset at the news (but) he didn’t baulk at doing the discontinuation questionnaire at all. |
| C | It would depend probably on the patient themselves. Some of them would really want to do it. I think that all has to do with personality and how they live their lives. If they’re committed to something they’re committed to something . . . I think it’s all personality dependent. I really do. |
| D | Is it your upbringing, background, general beliefs? I think it all has to do with it. |
| E | Depends on the shape of the person . . . some patients are a lot more stressed out than other patients, just dealing with [long pause of three seconds] the whole experience with cancer. |
| F | I’ll give you a a solid experience that I’ve had. He fell under standard arm, didn’t fall on the treatment arm which was immunotherapy. We offered him the quality of questionnaire on cycle one day one. He developed severe toxicities from the regular chemo: low platelets, absolute neutrophil count went down, he had **no** energy. After our second or third attempt to try to make sure that his uhm labs would resume so he could continue he ended up having a global deterioration and felt worse and worse. I went up to the department to see the patient. I asked him. I’ll leave this with you so you can complete it when you have a chance. I went back upstairs. It’s not that he didn’t want to he didn’t have the energy, was quite weak. I said “you know if you want I can ask you the questions and you’ll just answer the question and give me the answers”. He was quite advanced. He was trying his last chance at something. He completed it, made me repeat questions sometimes. He just didn’t want to have to [pause] go ahead and do it himself. I didn’t mind to have to uhm to read it to him. |
| I | There’s certain people for sure who are more likely to complete them. I think it depends on the patient. Most people [pause] are okay with doing the questionnaires. |
